# The Regulatory Approach for Faecal Microbiota Transplantation as Treatment for *Clostridioides difficile* Infection in Italy

**DOI:** 10.3390/antibiotics11040480

**Published:** 2022-04-05

**Authors:** Maria Chiara de Stefano, Benedetta Mazzanti, Francesca Vespasiano, Letizia Lombardini, Massimo Cardillo

**Affiliations:** Italian National Transplant Centre, Istituto Superiore di Sanità, 00161 Rome, Italy; benedetta.mazzanti@iss.it (B.M.); francesca.vespasiano@iss.it (F.V.); letizia.lombardini@iss.it (L.L.); massimo.cardillo@iss.it (M.C.)

**Keywords:** *Clostridioides difficile* (*C. difficile*), faecal microbiota transplantation, FMT, FMT regulation, regulatory framework, EU Tissue and Cell Directive

## Abstract

Faecal microbiota transplantation (FMT) is regarded as an efficacious treatment for recurrent *C. difficile* infection. Unfortunately, widespread patient access is hindered by regulatory hurdles, which are the primary barriers to incorporating FMT into clinical practice. At the European and International level, there is no uniform perspective on FMT classification, and a coordinated effort is desirable to solve this regulatory puzzle. In this communication, we report the regulatory principles and the implementation approach for FMT application in Italy. Our experience suggests that the EU Tissue and Cell Directives are suited to ensure safe and efficient FMT for *C. difficile* management, especially through extensive high-quality donor selection and full traceability maintenance.

## 1. Regulatory Approaches for Faecal Microbiota Transplantation in *Clostridioides difficile* Infection (CDI) Management

Disease recurrence is a relevant issue in CDI management, as up to 25% of patients experience at least one recurrence within 8 weeks of successful antibiotic therapy for the initial episode, and a first recurrence, in turn, increases the risk of subsequent recurrences [1,2]. This scenario can negatively impact the rate of hospitalization and patient survival as well as the clinical and healthcare burden [1,3,4]. In addition to risk factors such as advanced age, immune status, comorbidities, and continued antibiotics use, poor bacterial diversity of gut microbiota has been implicated in the development of recurrent CDI infection (rCDI) [2,5]. Unfortunately, antibiotics treatment for CDI, such as vancomycin and fidaxomicin, can affect the function and reduce the overall species diversity of gut microbiota, predisposing patients to rCDI and leading to a vicious cycle of recurrent disease [2]. A large body of evidence supports the role of faecal microbiota transplantation (FMT) in restoring colonization resistance to *C. difficile* [6,7,8], and such a therapeutic approach is recommended for patients with multiple CDI recurrences after failing antibiotics therapy by the European Society for Microbiology and Infectious Diseases as well as the American College of Gastroenterology [9,10]. As the term implies, faecal microbiota transplantation consists of transferring a faecal sample from a healthy donor into the gastrointestinal tract of a patient. A recent survey reported that approximately 2000 hospital-based FMT procedures were performed across European countries in 2019, with most referred to CDI clinical indication [11]. Despite the growing demand for FMT for patients with multiple recurrences of CDI, its incorporation into clinical practice remains a challenging task [11,12]. Indeed, no uniform perspective on FMT classification exists, and the regulatory authorities have developed different approaches to oversee the clinical FMT framework. The U.S. Food and Drug Administration (FDA) considers faecal microbiota a new biological drug, even though the introduction of enforcement discretion allows FMT use without an Investigational New Drug Application (IND) to treat *C. difficile* infection not responding to standard therapies [13]. In the U.K., FMT was initially regulated under the Human Tissue Authority, but it has been falling under the definition of a medicinal product and into the remit of the Medicines and Health care products Regulatory Agency since 2015 [14]. The U.K. change of perspective reflects the ongoing uncertainty surrounding the regulatory framework for faecal microbiota, considering that little is known about the active component and intrinsic mechanism of the action of FMT. The classification as a drug implies a strict monitoring of microbiota processing since the stool would be industrially manufactured in a batch-wise process to be placed on the market [15]. However, a stool is not a standardized mixture of bacteria, and the composition of a stool by itself is highly heterogeneous, donor-specific, and associated with significant day-to-day variability, even from a single individual [14,16]. Moreover, it is likely that a positive effect on FMT success rate in rCDI is given by the transfer of a complete faecal microbiome rather than specific bacterial strains [17]. Overall, high- microbial diversity and the balanced constitution of Bacteroidetes and Firmicutes of the donor stool correlate with improved FMT efficacy [18]. It is known that *C. difficile* colonization is associated with a marked decrease in Bacteroidetes and Firmicutes and a high increase in Proteobacteria [19]. Interestingly, FMT shifts the patient faecal microbiota profile to that of the healthy donor with relative reductions in Proteobacteria and relative increases in Bacteroidetes and Firmicutes [20]. Therefore, FMT reverses the dysbiotic state, restoring the normal composition of gut microbiota, and so it also has a significant impact on the host immune system and the metabolism of secondary bile acids, which can inhibit *C. difficile* germination and vegetative growth [19]. However, the underlying mechanism for FMT remains not fully understood, and further investigation is needed for the development of standardized microbiota replacement therapies.

In this context, the European Commission has left decision making in the hands of the individual Member States and left them free to decide on the most suitable framework at the national level, even though several European countries released no official determination on FMT regulation. Currently, some European Member States such as the Netherlands, Belgium, and Italy, have included faecal microbiota under the tissues and cells regulation, while in other countries, including France and Germany, stool has been classified as a drug [14]. However, it is worth mentioning the clear position of the United European Gastroenterology-funded working group on this issue, as it considers the stool a transplant product and demands that competent authorities supply a comprehensive regulatory framework to ensure FMT broad access and safety [21]. Accordingly, the Italian National Transplant Centre (CNT) has been leading the Italian National FMT Program for three years, addressed to all Italian FMT centres, with the aim of coordinating and standardizing the clinical framework for the application of FMT in rCDI.

## 2. Principles and Implementation of the Italian Regulatory Approach for FMT

**Figure 1:**
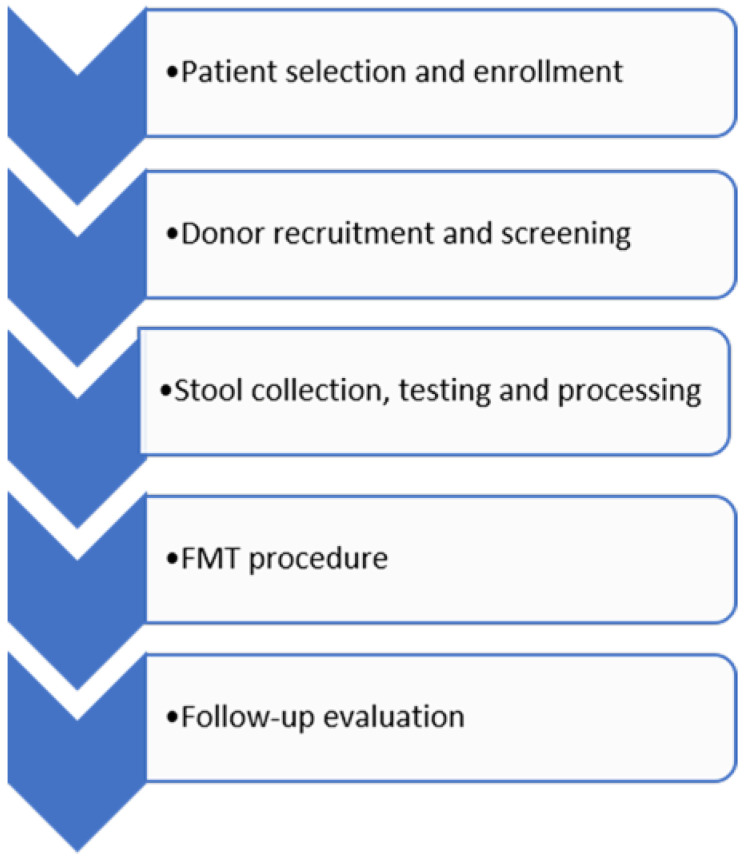
FMT process basic flow chart. The main sequential steps are represented.

The major challenges for the application of FMT as a treatment for rCDI are achieving well-defined safety and quality standards as well as ensuring FMT availability for patients. Many of the potential safety risks are linked to the donor selection and screening process, as an important safety alert about the transmission of multi-drug-resistant organisms through FMT has recently been released [22]. Therefore, stool samples need to be tested for antibiotic-resistant bacteria including methicillin-resistant Staphylococcus aureus (MRSA), vancomycin-resistant Enterococci (VRE), extended-spectrum β-lactamase-producing Enterobacteriaceae, and carbapenem-resistant Enterobacteriaceae/carbapenemase-producing Enterobacteriaceae. In Italy, all steps from donation to patient follow-up, including donor’s selection and stool processing, must follow the stringent safety and quality requirements in compliance with Directives 2006/17/EC and 2006/86/EC implementing the European Union Tissues and Cells Directive (EUTCD) on the quality and safety of tissues and cells. In particular, Directive 2004/23/EC (EUTCD) lays down standards of quality and safety covering the donation, procurement, testing, processing, preservation, storage, and distribution of human tissues and cells [23]. In addition, specific Italian National guidance providing technical information on how to apply FMT in rCDI setting was issued, and the key characteristics have already been described [24]. As shown in Figure 1, the FMT process consists of sequential steps, from the patient selection to the follow-up evaluation, and it is based on a systematic and multidisciplinary approach requiring the involvement of an expert panel of microbiologists, gastroenterologists, and infectious disease specialists. In order to establish an FMT service, appropriate operational procedures must be in place.

Table 1 describes the measures required for FMT, as provided for the Italian National FMT Program and in accordance with EUTCD and the recommendations published by the European Directorate for the Quality of Medicines and HealthCare (EDQM) of the Council of Europe [25].

In particular, a quality system ensuring minimal risk for product, personnel, donor, and patient and maximal quality of the process shall be developed by the FMT centre, including an organizational chart with a clearly stated hierarchy of duties and responsibilities of the personnel involved. Personnel education and training need to be ensured and traced by specific procedures and records. Standard Operating Procedures (SOPs) for each step of the process shall be in place and periodically updated. According to Directive 2004/23/EC, a system shall be implemented with the aim to register and transmit to the Italian National Competent Authority (i.e., CNT) the information about serious adverse events and reactions influencing the quality and safety of FMT and attributable to the procurement, testing, processing, storage, distribution, and clinical application. The procurement of faecal microbiota shall be authorized only after informed consent of the donor once adequate information has been given. Likewise, consent approved by the local ethics committee shall be provided to the patient undergoing FMT. Donor recruitment and screening shall be carried out in compliance with the Italian National FMT program recommendations and in accordance with the European consensus guidelines [26]. Of note, the procurement procedures must preserve those properties of the stool material that are required for their ultimate clinical use and, at the same time, should be addressed to minimize the risk of microbiological contamination during stool collection and processing. Preferably, a dedicated bathroom should be used, or detailed instructions shall be provided to the donor in case of collection at home. In addition, the processing facility should provide sterile faecal containers in order to prevent contamination, and the stool preservation condition should be strictly monitored. The donor screening tests must be carried out by authorized and qualified laboratories, using EC-marked testing kits where appropriate, and be validated for the purpose in accordance with current scientific knowledge. Regardless of the findings, all results of the donor evaluation and testing procedures shall be documented. In order to guarantee the quality of stool handling, the laboratory is required to define critical parameters affecting the stool processing and the viability and composition of the microbial content. Moreover, a biosafety Level 2 processing facility is needed, and all personnel involved in processing activities must be trained and provided with protective clothing appropriate for the type of processing, wearing a sterile gown, sterile gloves, glasses, and a face shield or protective mask. Critical reagents and materials must meet documented requirements and specifications and, when applicable, should be single-use and disposable. The frozen faecal material should be stored in dedicated freezers under controlled storage conditions, subjected to appropriate monitoring, and provided with alerts for temperature. Maximum storage time shall be defined and validated by counting the microbial load of defrosted aliquots compared to the fresh preparation [27]. For each critical activity, the materials, equipment, and personnel involved must be identified and documented. The materials traceability shall be assured by recording data on materials and reagents (e.g., name of product and producer, lot, expiry date, and results of internal quality controls) used for the processing, and quality tests shall be successfully passed for the release of the final product. Overall, it is the responsibility of the processing laboratory to confirm final product compliance with the quality and safety requirements prior to distribution and administration. Finally, the implementation of the above-mentioned principles is a preliminary condition so that an FMT centre can be subjected to auditing by the CNT for the authorization purpose. Before applying for authorization, the FMT centre should review each step of the process to identify and improve all critical issues and procedures failing to comply with requirements of EU Directives/national law as well as the EDQM recommendations. Of note, the missing knowledge of the EU Directives requirements and the poor understanding on how to set out the SOPs often hamper and postpone the finalization of the accreditation process. More worryingly, a few centres are not fully aware that they have to report to the National Competent Authority for clinical FMT application. With the aim of supporting healthcare professionals at a practical level and promoting efficacious and safe clinical application, the CNT provides rules and organizational support to centres committed to developing a local clinical FMT framework in adherence to the European Tissue Act. Moreover, in order to foster continuous harmonization and to prevent fragmentation following different local approaches, the CNT promotes collaborative knowledge exchange through periodic virtual and face-to-face meetings as well as dissemination and training activities. However, it is worth noting that some differences remain regarding the organization of donor recruitment, the laboratory facilities and processing procedures. To monitor the overall functioning of the FMT centres network, the data of the centres’ activities are collected in a dedicated database including quality and performance indicators such as the number of procedures performed according to Italian National FMT standards, transplant outcomes, any serious adverse events and/or reactions, as well as long-term follow-up evaluation. Currently, 57 patients (Female = 27, Male = 30; median age 70 years) have undergone FMT with faeces from 11 donors (Female = 8, Male = 3; median age 41 years). Overall, 68 transplants have been collected, as all patients were treated with at least one infusion, and 14 patients received multiple infusions. According to Ianiro G. et al. [28], our data show that sequential FMT is highly effective (success rate of 83% with single infusion versus 100% with sequential FMT). Notably, no serious adverse events and/or reactions were notified.

In order to gain insight into challenging issues, such as the FMT application in paediatric patients and in non-CDI clinical settings, a multidisciplinary network of experts in new potential indications for FMT is being established. Experts’ participation in decision making will be strongly endorsed so that high-level evidence may be integrated more easily into clinical practice and translated into direct benefits for patients in terms of enhanced quality of care and safety. Meanwhile, to identify any patient safeguarding concerns, FMT in non-CDI indications must be conducted under a clinical trial to be submitted to the CNT for approval [24].

## 3. Conclusions

The FMT has been evaluated as a cost-effective strategy for the management of rCDI, with considerable cost savings mainly achieved by a significant reduction in the total days of hospital admission due to a faster recovery time [29]. Furthermore, emerging data support the intermediate to long-term safety profile of FMT for rCDI [30,31]. Interestingly, it has also been reported that FMT treatment could prevent as many as 32,000 CDI recurrences every year in the United States [32]. Therefore, the scale of FMT use may be increased to meet the significant need for patients with rCDI. Unfortunately, widespread access to this therapeutic option is hindered by regulatory frameworks’ lack of uniformity, which is the primary barrier to embedding FMT into routine patient care. On one hand, faecal microbiota does not fall within the statutory scope of Directive 2004/23/EC on Tissue and Cells legislation because, although faeces are unquestionably substances of human origin, the human cells do not represent the active component [33]. On the other hand, the variability in gut microbial community composition and across stool samples does not meet the requirements of a batch-wise manufacturing process underlying the classification as a drug [15,34]. However, Member States’ competent authorities agreed that FMT should be regulated by provisions equivalent to those existing for blood, tissue, and cells, because the FMT entails similar risks, including disease transmission [33]. For this reason, the Italian approach has been addressed to incorporate EUTCD and EDQM recommendations within hospital settings, supporting the development of FMT centres complying with high safety and quality standards. In order to provide ready-to-use screened faeces preparations and to facilitate patient access to the FMT treatment, specific transplant programs, including stool banks, will be set up. To this aim, detailed stool banking guidance is being developed at a national level, in accordance with European and International consensus reports [35,36]. Our model provides a FMT transplant framework including a stool bank responsible for the processing, storage, and distribution of faeces preparations to clinical centres, as well as additional infrastructures needed for donor recruitment, selection, testing, patient treatment, and follow-up. In terms of feasibility, all the facilities may be part of the same organization or placed in different hospitals, under the responsibility of a qualified director who coordinates the activities according to shared, approved, and validated protocols. Overall, the development of strictly regulated FMT transplant frameworks is pivotal for increasing the cost effectiveness and coordinating an FMT transplant service by standardized pathways. Improving the standardization of the procedures is crucial, as different regulatory frameworks of FMT across Europe have a negative impact on equitable, safe, and timely treatment. We agree with Keller J.J. et al. that FMT should be regarded as a transplant and covered by the EUTCD because donated faeces are not subjected to substantial modifications prior to administration [21]. Furthermore, as reported above, the EUTCD implementation ensures an extensive high-quality donor selection and testing as well as monitoring of the traceability and potential risk profile changes.

In conclusion, it has clearly emerged that there is a need for increased harmonization in FMT regulation among Member States. The achievement of a coordinated European approach, including establishing a custom regulatory solution, will contribute to the large-scale accredited use of FMT for patients with rCDI.

## Figures and Tables

**Table 1:** Minimum requirements for FMT centres according to the Italian National FMT Program, EUTCD, and EDQM recommendations (4th edition).

EU Legislation	EDQM Guidance Principles	Italian National FMT Program Implementation Approaches
EC/23/2004 Art. 11;13;16;17;18 EC/86/2006 Art. 3;5;6EC/17/2006 Art. 2	Integrate FMT into a quality management system	- Use detailed written SOPs for each FMT activity (quality system in place). - Appoint a qualified responsible person and organizational chart which clearly defines roles and responsibilities in the FMT process. - Ensure continuous training and competency assessment of personnel involved. - Register the performance of all processing steps including the follow-up data. - Record any deviation, adverse events, and reactions. Notify the CNT of any serious adverse events and/or reactions. - Develop and use informed consents for both donors and patients according to National and International standards.
EC/23/2004 Art. 12;13;14;15 EC/17/2006 Art. 3;4;5	Supply high-quality donor selection and testing	- Provide a standardized, three-step donor selection process, including a written questionnaire to assess medical history and lifestyle habits to exclude risk factors for infectious diseases, blood and stool testing for any potentially transmittable disease, and a further questionnaire and stool testing the day of donation. - Obtain and maintain the European (or equivalent) accreditation for microbiological testing. - Verify the blood and stool screening testing acceptability. - Develop formal procedures for minimizing the risk of cross- contamination for donating, including establishing a dedicated donor bathroom and utilizing sterile containers and utensils.
EC/23/2004 Art. 19;20;21;22;23;28 EC/86/2006 Art. 3;4	Guarantee the quality and safety of stool processing	- Identify a processing workspace within a Level 2 biosafety laboratory. - Adopt operating procedures for stool handling complying with European and National guidelines, in accordance with scientific and technical progress. - Validate and maintain the equipment and materials as well as any critical manipulation step influencing the quality and safety of the product. - Use single-use sterile consumables and reagents, when possible. - Specify expiry timescales for the stool storage, counting the microbial load of the fresh preparation compared to defrosted aliquots.
EC/23/2004 Art. 8; Art. 10;25 EC/86/2006 Art. 9;10	Ensure full traceability	- Develop an electronic central system of recording and labelling, ensuring full traceability from donor to recipient and vice versa. - Provide the following processing records: unique donor identification number, donor testing results, date and time of donation, identification of recipient, donation macroscopic features, consumable and reagent lot numbers and volumes used, operator name, date and time of stool manipulation, storage instructions, and expiry date. - For every critical activity, the materials, equipment, and personnel involved must be identified and documented. - Provide a formal written statement confirming the final FMT product compliance with quality and safety requirements prior to administration.
